# Application of Aptamer-Based Biosensor for Rapid Detection of Pathogenic *Escherichia coli*

**DOI:** 10.3390/s18082518

**Published:** 2018-08-01

**Authors:** Yu-Wen Zhao, Hai-Xia Wang, Guang-Cheng Jia, Zheng Li

**Affiliations:** 1 College of Pharmaceutical Engineering of Traditional Chinese Medicine, Tianjin University of Traditional Chinese Medicine, Tianjin 300193, China; Zhaoyuwen_tju@163.com; 2 Modern Industrial Technology Research Institute of Traditional Chinese Medicine, Tianjin University of Traditional Chinese Medicine, Tianjin 300193, China; 3 Tasly Pharmaceutical Group Co., Ltd., Tianjin 300410, China; jia_guangcheng@163.com; 4 Tianjin Modern Innovation TCM Technology Co., Ltd., Tianjin 300410, China

**Keywords:** aptamer, sensor, *Escherichia coli*, rapid detection, electrochemical detector

## Abstract

Pathogenic *Escherichia coli* (*E. coli*) widely exist in Nature and have always been a serious threat to the human health. Conventional colony forming units counting-based methods are quite time consuming and not fit for rapid detection for *E. coli*. Therefore, novel strategies for improving detection efficiency and sensitivity are in great demand. Aptamers have been widely used in various sensors due to their extremely high affinity and specificity. Successful applications of aptamers have been found in the rapid detection of pathogenic *E. coli*. Herein, we present the latest advances in screening of aptamers for *E. coli*, and review the preparation and application of aptamer-based biosensors in rapid detection of *E. coli*. Furthermore, the problems and new trends in these aptamer-based biosensors for rapid detection of pathogenic microorganism are also discussed.

## 1. Introduction

*Escherichia coli (E. coli)* is one of the pathogenic bacteria that are widely spread in Nature. Since the middle of the last century, it has been recognized that some special serotypes of *E. coli* can produce enterotoxins, which can cause abdominal pain, diarrhea, inflammation, ulcers and even severe cases like hemorrhagic enteritis and hemolysis, especially in infants and young children [1,2]. Therefore, quantitative detection of *E. coli* has always been an important task in the field of environment, medicine, pharmacy and food safety. The Chinese national standard stipulates that pathogenic *E. coli* cannot be detected in the microbiological examination of drugs, which puts extremely high requirements on the detection limit of *E. coli* in practical applications. At present, there are three main types of detection methods for *E. coli*, i.e., traditional culture counting methods, molecular biology detection methods, and immunological detection methods. Traditional culture detection [3] is a method based on observing bacterial colony morphology, color change, and biochemical reactions in a specific medium after the isolation and culturing of bacteria. It can be divided into two types: the plate counting method and the most probable number (MPN) counting method. The plate counting method can be used to detect plates with colonies ranging from 15 to 150 colony forming units (CFU), while the MPN counting method is suitable for the counting of coliforms in samples with lower coliform content. Owing to their high credibility, low detection limit and simple operation, they have been regarded as the golden standards for *E. coli* detection. However, the application of traditional culture methods is severely limited due to their long detection time (72–120 h). Compared with the traditional methods, the polymerase chain reaction (PCR) technique has been widely used for *E. coli* determination [4,5]. The basic principle of PCR is that DNA templates are denatured by high temperature in vitro, annealing and extension (one cycle) steps yield amplification. As the template DNA amplification product increases exponentially, increasing the number of cycles by 25–30 cycles can magnify the target segment by several million-fold, which greatly improves detection sensitivity [6]. However, humic acid, being a PCR inhibitor, is more likely to produce false negatives [7], which affects PCR extensive application. In addition, enzyme linked immunosorbent assay (ELISA) is also a candidate method for *E. coli* detection. ELISA method relies on the ability of immunological binding reaction between an antigen and an antibody. By labeling the tracer on the reactant, the amount of antigen or antibody can be analyzed and detected in the experiment [8]. Due to the combination of specificity of antigen and antibody immunoreactivity, and the efficiency of enzyme catalysis, the ELISA method has good specificity and sensitivity. In addition, the detection time of ELISA method based on rapid binding of antigens and antibodies is greatly shortened. Therefore, the analysis and quantification of *E. coli* in water and food by ELISA method has received widespread attention [9,10]. However, the shortcomings of ELISA method cannot be ignored, e.g., it is difficult, antibody preparation takes a long time and it has a high detection limit in protein analysis [11,12]. Moreover, some samples with small molecular weight have no immunogenic activity, and need be coupled to macromolecular proteins for ELISA detection.

Aptamers are single-stranded oligonucleotides obtained by a systematic evolution of ligands by exponential enrichment (SELEX) technique [13] and have high affinity [14] to target molecules, such as proteins, amino acids, pathogens, viruses, peptides, and even cells. Due to their advantages of easy synthesis and modification, and high stability, affinity and specificity, aptamers have been widely used in the detection of biotoxins [15], heavy metal ions [16], pesticide residues [17] and other harmful substances [18,19]. In recent years, *E. coli* detection based on specific aptamers has attracted more and more attention [20,21]. Li and his group reported a method for rapid detection *E. coli* using an aptamer with fluorescence catalytic ability as a detection probe, the detection time of which is only half an hour [22]. Fu linked the aptamer to an optical sensor that provided a visual inspection method for *E. coli*, the detection sensitivity could reach 10 CFU/well [23]. Malhotra’s aptamer-electrochemical sensor can simultaneously detect *E. coli* DNA and *E. coli* cells with detection limits of 0.01 ng/μL and 11 CFU/mL, respectively [24]. In addition, the introduction of nanomaterials [25], graphene [26], carbon nanotubes [27] and other new materials have provided further advances in *E. coli* detection, which can greatly improve the effectiveness, selectivity and sensitivity.

Although there have been some comprehensive review articles published aiming to illustrate the latest progress on biosensors for the detection of foodborne pathogens [28,29], reviews on aptamer biosensors for the detection of *E. coli* are relatively rare [30,31], and few are about fast detection. Therefore, we present this review focusing on the rapid detection of pathogenic *E. coli* with aptamer biosensors, including the screening of aptamers for pathogenic *E. coli*, detection sensors based on *E. coli* aptamers, and the sensor application in *E. coli* detection. The discussions include the problems in current research and the future of these aptamer-based biosensors for rapid detection of pathogenic *E. coli*.

## 2. Screening of Pathogenic *E. coli* Aptamers by SELEX Technique

**Scheme 1:**
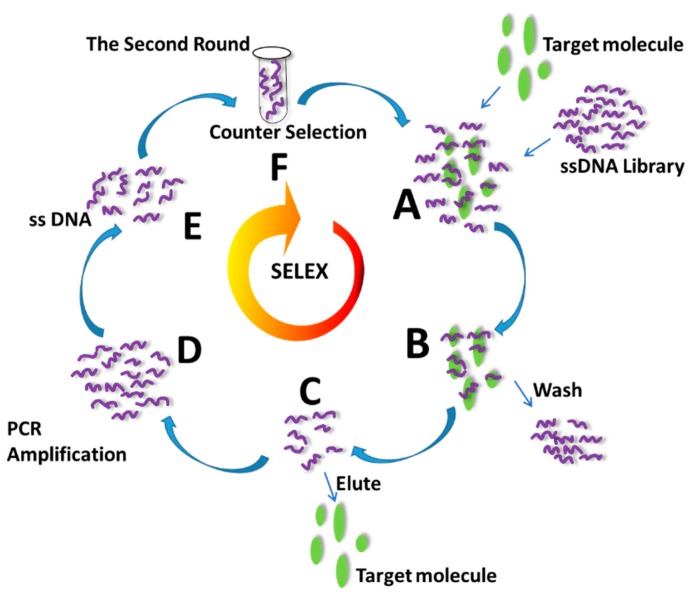
Schematic representation of the aptamer selection.

SELEX technique is a generic method for aptamers screening. The most critical step in the SELEX process is the selection and separation of the nucleic acid sequences that are binding and non-binding to the target molecule. The targeted nucleic acid sequences binding to a target molecule are found by mixing the target molecule with a large pool of random single-stranded DNA or RNA libraries. After washing off oligonucleotides that are not bound to a target substance, the high-specificity and high-affinity nucleic acid sequences bound to a target molecule can be separated. Using these nucleic acid molecules as a template, PCR amplification is performed and the next round of screening and amplification process are repeated until the aptamer is found. Scheme 1 shows a traditional schematic representation of the aptamer selection. It is worth mentioning that the traditional SELEX process takes a long time. Therefore, in recent years, a series of emerging SELEX technologies have been developed for reducing screening time or improving binding ability to target molecule, e.g., the whole bacteria-SELEX technique [32], the subtraction SELEX technique [33], the cell-SELEX technique [34], etc.

The main advantage of cell-SELEX technique is that the selected multiple aptamers that could potentially bind to different targets on the cells in their native conformation and physiological environment, without protein purification before selection [20]. Marton [20] used cell-SELEX technology to screen out four aptamers to *E. coli* with dissociation constants in the nanomolar range. Furthermore, they selected 12 different *E. coli* species for aptamer specificity tests. Fluorescence analysis showed that one of the aptamers was associated with meningitis/septicemia-associated *E. coli*, which has positive guiding significance for the treatment of meningitis/septicemia. Nasa [34] reported a new technique by combining the aptamer conformational analysis with the quantitative PCR-controlled cell-SELEX technique, which greatly reduced the number of SELEX cycles and improved the aptamer selection accuracy. In order to compare the affinity and specificity for urinary pathogenic *E. coli* of the obtained 29 aptamers, they initially examined the homology of aptamers. However, probably owing to the complex structures of cell surface that presents multiple binding targets, the obtained 29 aptamers lacked identical or highly similar sequences. Afterwards, they characterized aptamers in terms of putative secondary conformations, and the presence of sequence motifs predicted to form a variety of structures, four of which contained G4-motifs. The results also indicated that their qPCR-controlled cell-SELEX preferentially enriched G4-forming aptamers for urinary pathogenic *E. coli.* Based on this method, a new DNA aptamer with high affinity and specificity for urinary pathogenic *E. coli* was screened, which was of great significance for the rapid diagnosis and treatment of urinary tract infection caused by *E. coli*. Lee et al. screened a new RNA aptamer sequence with high recognition specificity using the subtractive cell-SELEX method [33]. Rather than conventional SELEX approach using crude or purified extracellular matrix as target molecules, their cell-targeting SELEX technique was employed for the selection of an aptamer specific to the *E. coli* O157:H7 strain surface. The aptamer screened by this method specifically recognized enterohemorrhagic *E. coli* O157:H7 but not K-antigen type. Importantly, the RNA aptamer distinguished between the pathogenic and the nonpathogenic enterohemorrhagic *E. coli* that includes the O antigen. In addition, in order to eliminate the need of purifying target molecules on the cell surface, Wang screened two stable aptamers specific to the *E. coli* by using the whole bacteria-SELEX technique. This technique used live bacterial cells in suspension as targets to select single strand DNA (ssDNA) aptamers specific to cell surface molecules. The screened two aptamers have high affinity for enteropathogenic, enterohaemorrhagic and enterotoxigenic *E. coli*, but not others [35]. Table 1 summarized the different pathogenic *E. coli* aptamers in recent years, including the sequences, recognition sites, and target strains of *E. coli*.

## 3. Aptamer-Based Optical Sensors for Pathogenic *E. coli* Detection

The aptamer-based optical sensors, including visible light, ultraviolet, and fluorescent sensor, have been developed and widely used. Because of the advantages of easy operating and cost saving, the visual detection of *E. coli* has been a goal of biological and analytical chemists. At present, ELISA techniques can visually detect *E. coli* [57,58]. However, it requires a large amount of labeled antibodies, a long detection time and a high detection cost. Visual aptasensors for rapid *E. coli* detection have been developed to meet these challenges.

Lv et al. constructed an aptamer-polydiacetylene (PDA) optical sensor for rapid colorimetric detection of *E. coli* O157:H7 [47]. PDA vesicles are a dark blue substance that has an UV-vis absorption peak at 640 nm. When the *E. coli* O157:H7 aptamer binds to PDA vesicle surface, a new absorption peak appears at 260 nm, proving the success of the aptamer-PDA optical sensor assembly. Afterwards, the UV-vis absorption peak at 640 nm blue shifted to around 540 nm owing to the molecular recognition between aptamer at the vesicle surface and *E. coli* O157:H7. The red-blue transition of PDA was readily visible and could be quantified by colorimetric responses (CR). The aptamer-PDA sensor can detect cellular concentrations in a range of 10^4^–10^8^ CFU/mL within 2 h and its specificity was 100% for *E. coli* O157:H7 detection. This study provided a novel colorimetric detection method for *E. coli* O157:H7, but the detection limit at 10^4^ CFU/mL is relatively high that needs to be improved further.

A method that could rapidly and visually detect *E. coli* K88 was established based on the sandwich assay. Biotin-labeled aptamer 1 that could specifically bind to *E. coli* K88 was incubated with target bacterium *E. coli* K88 and the Au nanoparticles (NPs)-labeled aptamer 2 conjugates at first, to form a sandwich-type biotin-labeled aptamer 1/*E. coli* K88/NPs-labeled aptamer 2 complexes. At the same time, the streptavidin-labeled plate was blocked nonspecific sites by bovine serum albumin (BSA). Then, the sandwich-type complexes were transferred onto the surface of the plate modified with streptavidin through the binding of biotin and streptavidin. Owing to the catalytic reduction activity of Au NPs, the silver ions were reduced to elemental silver deposited on the surface of the Au NPs, resulting in a distinct color reaction, thereby enabling the sandwich complexes to visually detect *E. coli* K88 at the peak of 630 nm with a linear response in the range from 10 to 10^5^ CFU/mL (Figure 3 in [23]). However, the stability and specificity of the aptamer sandwich composite should be further investigated in clinical context by this visual detection method for *E. coli* K88.

Compared to visual and colorimetric detection, *E. coli* aptasensors based on fluorescence measurement have many advantages in terms of detection sensitivity, specificity selection, etc. Duan et al. constructed an aptamer-fluorescent sensor by using two aptamers labeled with biotin and fluorescein amidite (FAM) probe, respectively. Biotin-labeled aptamer 1 was used as capture probe, which was immobilized onto the avidin-labeled plate through the specific binding of biotin and avidin at first. FAM labeled aptamer 2 was used as report probe. In the presence of target bacterium *E. coli*, the biotin-labeled aptamer 1 would capture and bind with *E. coli*. In addition, FAM labeled aptamer 2 would also bind with *E. coli* and displayed fluorescence signal. The prepared aptamer-fluorescent sensor has a good detection activity in the concentration range of 50–10^6^ CFU/mL for *E. coli* [36].

Li and his group [22] innovatively applied a fluorogenic DNAzyme (RFD-EC1) for selectively recognizing the crude extracellular mixture of *E. coli* K12. The addition of RFD-EC1 could cause dramatic increase in fluorescence signal after mixing with *E. coli* K12 extracellular matrix for 35 min. The subsequent specific experiment indicated that the RFD-EC1 is highly specific to the extracellular matrix of *E. coli* but not to the other 14 bacteria. The most attractive feature advantage of this method is that it does not need to consider the tedious isolation and identification of extracellular matrices steps, but just takes only two steps of “mixing” and “reading” to directly detect the fluorescence signal of the target microorganism. Moreover, the innovative method can detect single living cells directly. Since this method was proposed, it has attracted much attention and related improvement research is still in progress.

## 4. Aptamer-Based Electrochemical Sensors for Pathogenic *E. coli* Detection

Compared with optical sensors, electrochemical sensors [59,60,61] are easy to achieve miniaturization and portability, and can be used for turbid medium detection. Therefore, aptamer-based electrochemical sensors for pathogenic *E. coli* detection have also attracted much attention [62,63,64]. Electrochemical detection signals can be current, resistance, or potential, which provides great selectivity for electrochemical sensors testing. In addition, the emergence of more sensitive, miniaturized and intelligent working electrodes [65,66,67] has provided divergent choices for microbiological detection. As far as we know, the aptamer-electrochemical sensor can be used at gene, protein and cell levels for *E. coli* detection.

### 4.1. E. coli Genetic Testing by Aptamer-Electrochemical Sensor

**Figure 1:**
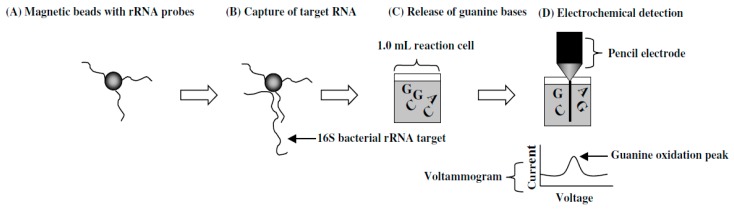
Outline of the electrochemical *E. coli* RNA hybridization assay. (**A**) DNA probe-labeled magnetic beads was obtained by conjugation of streptavidin-coated magnetic beads and biotin-labeled oligonucleotide probes; (**B**) *E. coli* rRNA and DNA probe-coated magnetic beads were ligated together to construct an rRNA-magnetic bead complex; (**C**) after washing with strong alkali and strong acid solution, the *E. coli* rRNA was separated from the magnetic beads and the free guanine nucleotides were then released; (**D**) the electrochemical oxidation signal of the released guanine nucleotides can be detected by pulse voltammetry at a pencil graphite electrode [71].

Electrochemical biosensors have been considered as a simple and sensitive method of DNA or RNA hybridization detection [68,69,70]. It has been reported to detect complementary sequences of *E. coli* DNA and RNA in a short period of time. LaGier et al. detected *E. coli* ribosomal RNA (rRNA) by monitoring of the oxidation state of guanine nucleotides [71]. The specific testing process is composed of several steps (Figure 1). Firstly, *E. coli* rRNA and DNA probe-coated magnetic beads were ligated together to construct an rRNA-magnetic bead complex. Secondly, after washing with strong alkali and strong acid solution, the *E. coli* rRNA was separated from the magnetic beads and the free guanine nucleotides were then obtained. Finally, the electrochemical oxidation signal of guanine nucleotides can be detected by using a specific pencil electrode [72]. Using this approach, *E. coli* rRNA content can be determined without a nucleic acid amplification step and 10^7^ cells of *E. coli* were detected in a quantitative manner directly. However, the further application of the method is subject to following factors: the high detection limit, the cumbersome RNA extraction steps and the expensive reagent cost.

**Figure 2:**
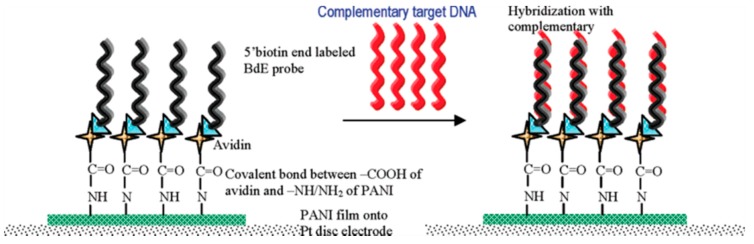
DNA hybridization detection using a PANI-Pt electrode. Biotin-labeled *E. coli* capture probe (BdE) was primarily immobilized on PANI-modified Pt disk by the covalent bond between –COOH of avidin and –NH/NH_2_ of PANI. A complemental *E. coli* genomic DNA sequence was introduced into the modified Pt disk by DNA hybridization subsequently [24].

Malhotra et al. prepared polyaniline (PANI)-modified Pt disk electrode for direct detection of *E. coli* genomic DNA by using methylene blue as a DNA hybridization indicator [24] (Figure 2). Biotin-labeled *E. coli* capture probe (BdE) was primarily immobilized on PANI-modified Pt disk by carbodiimide activation. A complementary *E. coli* genomic DNA sequence was then introduced into the electrode, the remaining single-stranded BdE can be specifically binding with methylene blue and the electrochemical signal can be monitored according to a differential pulse voltammetric (DPV) technique in 60 s to 14 min (hybridization time) [73]. This bioelectrode not only has a satisfactory detection limit for *E. coli* genomic DNA (0.01 ng/μL) and *E. coli* cells (11 CFU/mL), but it can be reused 5–7 times at 30–45 °C.

### 4.2. E. coli Protein Detection by Aptamer-Electrochemical Sensor

**Figure 3:**
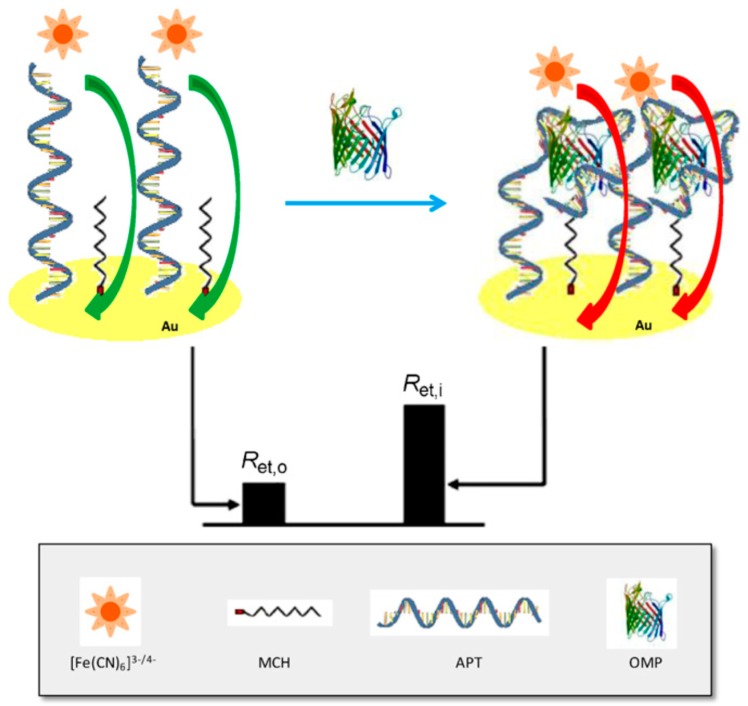
Schematic illustration of the modified electrodes and the detection of *E. coli* OMPs. APT refers to a thiolated OMPs aptamer [74].

A DNA aptamer-based impedance biosensor for the detection of *E. coli* outer membrane proteins (OMPs) was developed to identify membrane proteins with simple detection principle and easy-to-operate process [74] (Figure 3). A thiolated OMPs detection aptamer was immobilized on a gold electrode at first. A self-assembled monolayer formed by MCH (6-mercapto-1-hexanol) was then stacked onto the modified electrode surface to prevent nonspecific adsorption of the OMPs. In the presence of *E. coli* OMPs, the OMPs could be adsorbed on the modified electrode surface by the specific recognition with the OMPs aptamer. Using potassium ferricyanide as a redox probe, the added *E. coli* OMPs could hinder the electrons exchange between the redox probe and the electrode surface, and the electrochemical response can be recorded by faradaic impedance spectroscopy (FIS) technique. The proposed method has a high specificity and a good linear relationship between electron-transfer resistance (Ret) and the *E. coli* OMPs concentration in the range from 1 × 10^−7^ to 2 × 10^−6^ mol/L.

### 4.3. E. coli Cell Detection by Aptamer-Electrochemical Sensor

Although numerous aptamer-electrochemical sensors for gene and protein identification have been investigated with the purpose of *E. coli* rapid detection, it appears that the complex genetic and protein extraction steps and high reagent costs are still problems for practical detection applications. Therefore, an aptamer-electrochemical sensor for directly detecting *E. coli* cell with satisfactory detection limits and sensitivity but no pre-treatment steps is worth studying. Some researchers have been trying to select suitable aptamers and working electrodes in order to increase the accuracy and sensitivity of the electrochemical results in *E. coli* cell direct detection. The application of emerging aptamer screening techniques in *E. coli* aptamer selection has been described previously. Therefore, here we focus on the improvement of the working electrode in *E. coli* cell direct detection. As important electrode modification material, metal nanoparticles, graphene and single-walled carbon nanotubes (SWCNT) have been used in constructing electrochemical biosensors for improving the electron transfer and reducing the detection limitation [75,76,77]. Here, we present the latest advances for *E. coli* cell detection by using of electrochemical biosensors that combines metal nanoparticles, graphene and SWCNT.

**Figure 4:**
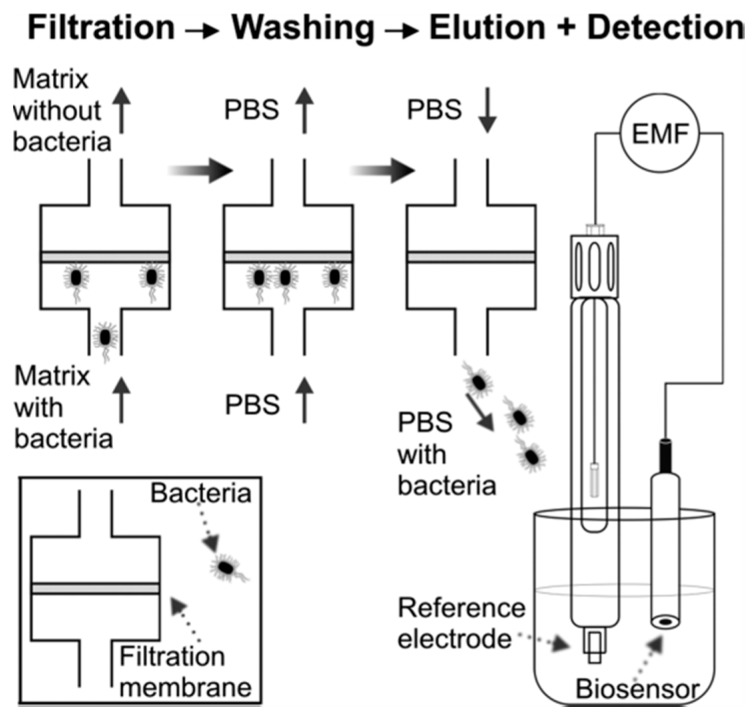
Testing procedure for real-time potentiometric detection of *E. coli* CECT 675. Starting from left to right: first step, filtration of sample and matrix removal; second step, washing with PBS; third step, elution with PBS and potentiometric detection of *E. coli* CECT 675 recovered in eluate [78].

As an excellent nanomaterial, SWCNT has a high specific surface area, and can greatly improve the surface adhesion and charge transfer rate. Riu [78] connected the *E. coli* CECT 675 aptamer with SWCNT through carbodiimide activation method. The aptamer/SWCNT/glassy carbon electrode (GCE) modified electrode were then constructed and used for *E. coli* CECT 675 cell on-line detection with potentiostat technology. Figure 4 shows the testing procedure of this method. Firstly, the macromolecular substances in the culture medium are removed by filtration. Secondly, the remaining charged substances in the bacterial broth are washed away with PBS. Finally, *E. coli* CECT 675 are resuspended in PBS and the electromotive force is real-time recorded. This method used an on-line filtration system that can perform real-time detection of *E. coli* cell in actual samples within minutes. The minimum detection limits in milk and apple juice were 6 CFU/mL and 26 CFU/mL, respectively. Moreover, the aptamer/SWCNT/GCE biosensor can be reused at least 5 times after electrode desorption operation. This technique is therefore a powerful tool for actual sample detection because of their low detection limit, short detection time and easily construction and regeneration capability.

**Figure 5:**
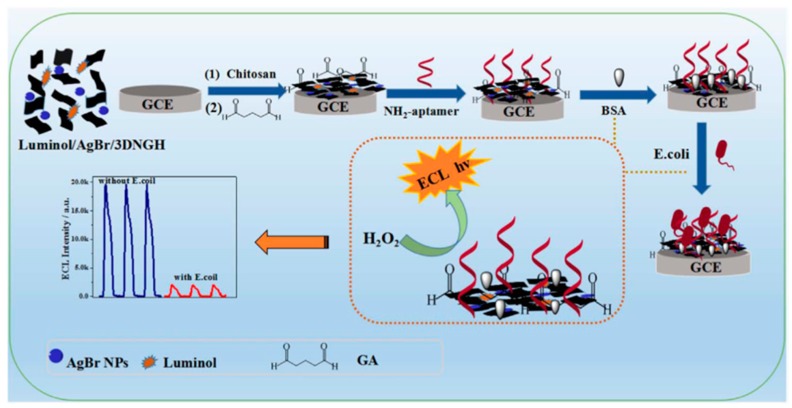
The schematic presentation for the *E. coli* biosensor fabricated with luminol/AgBr/3DNGH [79].

Hao et al. used AgBr NPs and a nitrogen doped three-dimensional graphene hydrogel (3DNGH) as electrode modification material for increasing loading rate and charge transfer rate [79] (Figure 5). The AgBr NPs anchored 3DNGH nanocomposites were prepared by hydrothermal approach at first. Then the luminol/AgBr/3DNGH/GCE modified electrode was obtained by drop coating method. In the third step, *E. coli* aptamer was immobilized on the luminol/AgBr/3DNGH/GCE modified electrode by using glutaraldehyde as a crosslinking agent. Additionally, BSA was used for blocking nonspecific sites of the modified electrode [80] to prevent nonspecific absorption of the *E. coli* and other impurities. Owing to the steric hindrance mechanism that *E. coli* can significantly decrease the electrochemiluminescence (ECL) intensity of luminol, the luminol/AgBr/3DNGH/GCE aptasensor displayed a linear response for *E. coli* in the range from 0.5 to 500 CFU/mL and the lowest detection limit was 0.17 CFU/mL. The extremely low detection limit may be ascribed to the better conductivity and higher loading rate of the luminol after introducing the AgBr/3DNGH composite materials with high specific surface area. However, the preparation process of this modified electrode is complicated and need to be simplified further.

**Figure 6:**
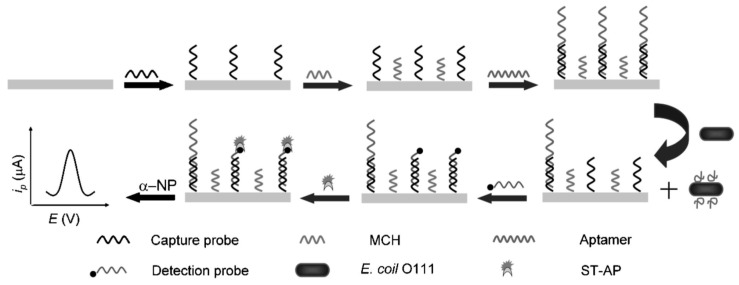
Principle of the electrochemical biosensor for detection of *E. coli* O111 [81].

Without using any other auxiliary modification materials, Luo et al. achieved the rapid detection of *E. coli* O111 solely with the aptamer-modified electrode [81] (Figure 6). They used three aptamer sequences for testing, capture probe, L9F aptamer and detection probe, wherein the L9F aptamer can specifically bind to lipopolysaccharide (LPS) on the membrane of *E. coli* O111. First, a thiol-modified capture probe that was in a complementary configuration to L9F aptamer was immobilized on the gold electrode by Au-S binding. After that, L9F aptamer was introduced into gold electrode by DNA hybridization. Due to the stronger interaction between L9F aptamer and *E. coli* O111, L9F aptamer can dissociate from the capture probe in the presence of *E. coli* O111. The biotinylated detection probe was then hybridized with the single-strand capture probe. After the modified electrode was washed with washing buffer, quantitative streptavidin-alkaline phosphatase (ST-AP) was dropped onto electrode surface and incubated at 37 °C for 30 min. As a result, the electrochemical response to *E. coli* O111 can be measured by using DPV in the presence of α-naphthyl phosphate. The plot of peak current vs. the logarithm of concentration in the range from 1 × 10^3^ to 1 × 10^6^ CFU/mL displayed a linear relationship with a detection limit of 305 CFU/mL in milk. Afterwards, their research group sensitized electrochemical signals by using exogenous exonuclease III and bfpA gene to reduce the *E. coli* detection limit to 50 CFU/mL and 10 CFU/mL, respectively [82]. Their process can achieve the *E. coli* rapid detection within 3.5 h. However, the detection process were cumbersome that need to be simplified.

**Figure 7:**
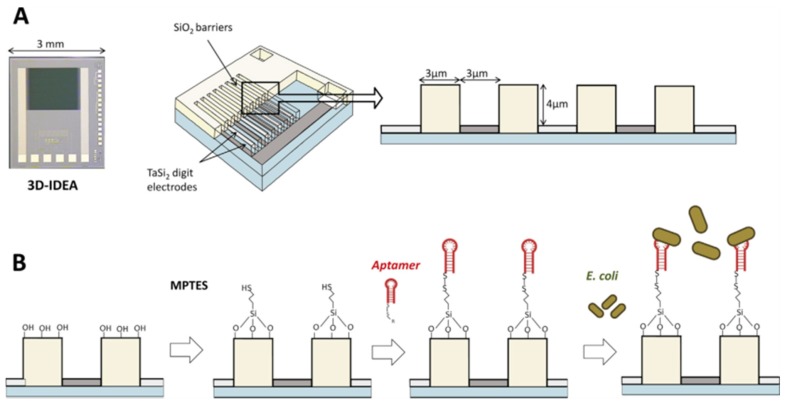
The schematic presentation of 3D-IDEA (**A**) and the different biofunctionalization steps for *E. coli* detection (**B**) [84].

The working electrodes of above research work were all single-point electrodes. Comparing to the traditional single-point bare electrodes, interdigital array electrodes (IDEA) have the advantages of smaller size, higher sensitivity, fewer sample volumes, and larger signal-to-noise ratio, and hence were widely used in biosensors for biomolecule detection [40,83]. However, there were few studies on aptamer-modified IDEA for microbiological testing. Recently, Bratov prepared aptamer-modified three-dimensional IDEA (3D-IDEA) and used them for *E. coli* rapid detection successfully [84] (Figure 7). 3D-IDEA was fabricated by using ultraviolet photolithography at first. Afterwards, the electrode surface was treated with coupling agent 3-mercaptopropyl-trimethoxysilane (MPTES). Then the *E. coli* aptamer probes were introduced into the surface of the 3D-IDEA through disulfide bonds between the terminal thiol groups of the coupling agent and the aptamer terminal chain. Due to the resistance mechanism that *E. coli* adsorbed on the surface of the 3D-IDEA blocks the free movement of electrons, the 3D-IDEA aptasensor displayed a linear response for *E. coli* in the range from 10 to 10^5^ CFU/mL. It only took a few minutes to detect *E. coli* cell concentration by using this 3D-IDEA aptasensor. The specific experiments showed that this sensor has a good anti-interference performance, and the 3D-IDEA aptasensor can be reused by heat treatment. The aptamer-modified IDEA aptasensor can be a new candidate for rapid detection of *E. coli* and is worthy of promotion and reference. However, the cost of this kind of IDEA electrode is relatively high for practical applications.

In addition, graphene paper-based biosensors, has been developed and used for *E. coli* rapid detection [85]. Pulsed sonoelectrodeposition technology was selected to immobilize nanoplatinum with broccoli configuration on grapheme conductive paper. Since the *E. coli* aptamers have been previously covalently linked to the nanoplatinum, *E. coli* can be loaded on the surface of the nanoplatinum-graphene paper aptasensor. Similarly, the concentration of *E. coli* cell can be recorded by impedance measurements. Such nanoplatinum-graphene paper aptasensor displayed a satisfying detection result, and the detection limit was as low as 4 CFU/mL, while the detection time was only 12 min. It may be ascribed to the excellent conductivity of graphene and the large specific surface area of platinum nanoparticles with broccoli configuration. The new working electrode aptasensor based on graphene material has good conductivity that can be widely used for the rapid detection of other microorganisms, which was another major advancement in the field of electrochemical biosensors.

## 5. Conclusions and Prospects

In summary, aptamer-based biosensors have been widely used in rapid detection of pathogenic *Escherichia coli* because of specific properties of aptamers such as low cost, easy synthesis, easy modification, good stability, wide target molecules, and high affinity. However, the types of aptamers specifically binding to different pathogenic microorganisms are still relatively limited compared with a wide variety of antibodies, which restricts the development of aptamer biosensors in the detection of pathogenic microorganisms. Therefore, it is necessary to improve aptamer screening efficiency, expand the range of aptamer recognition targets, and increase the binding capacity to target molecules in the future. Moreover, compared with optical strategy, electrochemical proposal is likely to be used in actual sample test by the special advantages of easy-to-realize miniaturization and portability and not to be affected by the color of the liquid in the measurement process. Therefore, the development of a simple, efficient and rapid aptamer-electrochemical sensor is still the focus of microbiological testing in future. In addition, actual samples often contain many kinds of pathogenic microorganisms, and how to achieve simultaneous detection of multiple pathogenic microorganisms in a short period of time is still a major challenge. This requires efforts both in the screening of aptamers and in the design of the sensors. It is also worth noting that the rational use of new technologies and new materials may amplify the detection signal, improve the detection efficiency and expand the measurement scope in microorganism rapid detection. With the continuous development of aptamer technology and chemical detection strategy, broader application of aptamer-based biosensors in the rapid detection of pathogenic microorganisms can be envisioned.

## Figures and Tables

**Table 1:** Literature reported aptamers for pathogenic *E. coli.*

No.	Name	Sequence (5′ - 3′)	Target Strains of *E. coli*	Recognition Sites	Ref.	Follow up Work
1	Seq.1	ACCAGTAGACTTTCAACTTTACTGCCATCGTGTGCCCTAA	Enteropathogenic *E. coli*	Whole bacteria	[35]	[36]
2	Seq.28	ACAGTGCTCGGGATATATCAATATGTCACCTCGGCTAATG	Enteropathogenic *E. coli*	Whole bacteria	[35]	[36]
3	aptamer37	GGAGACCGTACCATCTGTTCGTGGAAGCGCTTTGCTCGTCCATTAGCCTTGTGCTCGTGC	Enterotoxigenic *E. coli*	Pilin protein	[21]	[37]
4	I-1	UGAUUCCAUCUUCCUGGACUGUCGAAAAUUCAGUAUCGGGAGGUUACGUAUUUGGUUUAU	Enteropathogenic *E. coli*	Lipopolysaccharide (LPS)	[33]	[38]
5	EcA5-27	GGCATAGCTGCCGGGAGGGGGGGG	Urethropathogenic *E. coli*	Whole bacteria	[34]	/
6	E2	CCATGAGTGTTGTGAAATGTTGGGACACTAGGTGGCATAGAGCCG	*E. coli* KCTC 2571	Whole bacteria	[39]	[40]
7	E1	ACTTAGGTCGAGGTTAGTTTGTCTTGCTGGCGCATCCACTGAGCG	*E. coli* KCTC 2571	Whole bacteria	[39]	/
8	E10	GTTGCACTGTGCGGCCGAGCTGCCCCCTGGTTTGTGAATACCCTGGG	*E. coli* KCTC 2571	Whole bacteria	[39]	/
9	E12	GCGAGGGCCAACGGTGGTTACGTCGCTACGGCGCTACTGGTTGAT	*E. coli* KCTC 2571	Whole bacteria	[39]	/
10	20#	CGAACGAATATAATTATGGCGTCCCCGGGGTTTCG	*Enterohemorrhagic E.coli*	Outer membrane proteins (OMPs)	[41]	/
11	L1F	CGTCGCTATGAAGTAACAAAGATAGGAGCAATCGGG	Enteropathogenic *E. coli* O111:B4	LPS	[42]	[43]
12	Eco 4 Rev	ACGGCGCTCCCAACAGGCCTCTCCTTACGGCATATTA	*E. coli* strain 8739	OMPs	[44]	/
13	Eco 3 Rev	GTCTGCGAGCGGGGCGCGGGCCCGGCGGGGGATGCG	*E. coli* strain 8739	OMPs	[44]	[45]
14	/	GGGAGAGCGGAAGCGUGCUGGGUCGCAGUUUGCGCGCGUUCCAACUUCUCUCAUCACGGAAACAUAACCCAGAGGUCGAU	*E. coli* DH5α	Whole bacteria	[46]	[46]
15	E17F-37	ATCAAATGTGCAGATATCAAGACGATTTGTACAAGAT	*Enterohemorrhagic E.coli*	LPS	[47]	[45,47,48]
16	E18R-42	CCGGACGCTTATGCCTTGCCATCTACAGAGCAGGTGTGACGG	*Enterohemorrhagic E.coli*	LPS	[47]	[49]
17	S1	TGGTCGTGGTGAGGTGCGTGTATGGGTGGTGGATGAGTGTGTGGC	*Enterohemorrhagic E.coli*	Whole bacteria	[50]	[50]
18	Ec3	GCACGAAUUUGCUGUGUUUUUGGGGGGGUCGGGGAGUAUA	*E. coli* DH5α	Whole bacteria	[51]	[51]
19	EA	CCGGACGCTTATGCCTTGCCATCTACAGAGCAGGTGTGACGG	*Enterohemorrhagic E.coli*	Whole bacteria	[38]	[38]
20	AM-6	GGGTGATGGGTGCATGTGATGAAAGGGGTTCGTGCTATGCTGTTTTGTCTAATAATACTAGTCCTTGCCAAGGTTTATTC	*Enterohemorrhagic E.coli*	Whole bacteria	[52]	[53]
21	ETEC-1	CTATAACTTTACTCCTAAGAACCCAAACAACACACA	*Enterotoxigenic E.coli*	Whole bacteria	[54]	/
22	Aptamer1	CGCAGTTTGGGAAGGGTGATCGCACTATCAGAGGATTCCGTTCGG	*Enterohemorrhagic E.coli*	Whole bacteria	[55]	[56]

## Abbreviations

*E. coli*	*Escherichia coli*
CFU	Colony forming units
PCR	Polymerase chain reaction
ELISA	Enzyme linked immunosorbent assay
SELEX	Exponentially enriched ligand system evolution
LPS	Lipopolysaccharide
OMPs	Outer membrane proteins
PDA	Polydiacetylene
CR	Colorimetric responses
Au NPs	Gold nanoparticles
RFD-EC1	Fluorogenic DNAzyme
rRNA	ribosomal RNA
PANI	Polyaniline
BdE	Biotin-labeled *E. coli* capture probe
DPV	Differential pulse voltammetric
FIS	Faradaic impedance spectroscopy
Ret	Electron-transfer resistance
SWCNT	Single-walled carbon nanotubes
GCE	Glassy carbon electrode
3DNGH	Three-dimensional graphene hydrogel
ECL	Electrochemiluminescence
IDEA	Interdigital array electrode
3D-IDEA	Three-dimensional interdigital array electrode
